# Exploring the traditional foodways for nutritional well-being amongst vulnerable communities: Insights from Ho indigenous community of Jharkhand, India

**DOI:** 10.12944/CRNFSJ.12.2.14

**Published:** 2024-05-09

**Authors:** Ridhima Kapoor, Manisha Sabharwal, Suparna Ghosh-Jerath

**Affiliations:** 1 Department of Food and Nutrition and Food Technology, Lady Irwin College, University of Delhi, Sikandra Road, New Delhi 110001, India; 2 The George Institute for Global Health INDIA, New Delhi 110025, India

**Keywords:** Foodways, Food Beliefs, Indigenous Populations, Lifeways, Smallholder Farmers

## Abstract

Nutritional well-being of Indigenous Peoples is shaped by foodways through their relationship with culturally vital indigenous foods. An exploratory mixed-methods study was conducted among Ho community of Jharkhand to get an insight into their traditional foodways. Study sites included ten randomly selected villages from three geographically distant blocks of West Singhbhum, including Sonua, Khuntpani and Chakradharpur. Qualitative enquiries included focus group discussions and village transect walk interviews which captured diverse information including food access from natural sources, market access, livelihood sources, social and cultural norms around foods, environmental factors, hygiene and sanitation conditions, and community health. Seasonal market surveys (monsoon and winter) in ten local markets provided information on food diversity and local prices. The findings revealed that foodways of Ho community are based on foraging, hunting, and traditional farming through use of available ecosystem resources. However, their traditional foodways are under threats due to livelihood and nutrition transitions. They have unique food traditions and cultures, yet their meals lack variety in terms of the foods consumed. Their smallholder farming systems are not profitable, pushing them towards rural-to-urban migration. Thus, it is crucial to promote traditional foodways of Hos to support bio-cultural knowledge, food justice and nutrition in this community.

## Introduction

Foodways are the cultural, social, and economic practices around food, including its production, preservation, preparation, presentation, gathering, marketing (both buying and selling), and utilization.^1^ Different communities vary in their foodways, and thus, it could be used to gain insights into their ways of living.^1^ Indigenous Peoples, the earliest inhabitants of a region, have unique traditional food systems, that includes landforms, vegetation, watersheds and climate zones, and provide substantial proportion of the world’s food genetic resources.^2^ Indigenous Foods or IFs, derived from these food systems, are accessed from wild and cultivated food sources, and consumed by the indigenous communities in ways that are culturally acceptable and reflect their indigenous foodways.^2^ However, the indigenous communities remain nutritionally vulnerable and lead a suboptimal quality of life, due to their limited socio-political rights, extreme marginalization, geographic isolation, and poverty.^3^

The Indigenous People in India, identified as ‘Scheduled Tribes’ (according to Article 342 of Indian Constitution), constitute 8.6% of the total national population,^4^ and are traditionally inhabitants of areas surrounded by forests, hills, mountains, and deserts.^5^ They possess rich bio-cultural diversity and their foodways are heavily dependent on natural produce for sustenance and livelihood, utilizing various seasonal food items.^6,7^ Thus, indigenous communities are closely tied to their nature, and are usually engaged in farming, foraging and livestock rearing.^6,7^ One such indigenous group, the ‘Ho’ community, is the fourth most populous indigenous group in Jharkhand, representing 11% of the total indigenous population in the state.^4^ This community belongs to the Proto-Australoid racial group of India, and are predominantly forest dwellers and subsistence farmers living in West Singhbhum district, surrounded with rich biodiversity.^8^ Though there is lack of published literature on their nutritional status, the district level data from National Family Health Survey-4 ^9^ of India indicate a high prevalence of maternal and child malnutrition. Thus, despite living in a biodiverse region and accessing natural resources for sustenance, the nutritional status of Ho tribe remains poor. A similar situation exists among other indigenous communities of India who remain highly vulnerable owing to food insecurity, under-nutrition and ill health.^9^

Globally, Indigenous communities have witnessed a drastic decline in the integrity of their food systems.^10^ The United Nations Food and Agriculture Organisation has recently identified ‘traditional food and culture’ as an important component towards meeting the sustainable development goals by 2030;^11^ however, the conventional food-based solutions for marginalized communities have often undermined the more holistic approaches rooted in their culture and foodways.^1^ According to the conceptual definition of foodways, the physical and social contexts primarily influence the food preferences and food practices, which in turn determine the food choices and places of food access.^12^ The health and nutritional well-being of Indigenous Peoples, in particular, is shaped by foodways through their relationship with culturally vital IFs, including their production, harvesting, processing, cooking, serving, and consumption.^1^

While there has been increasing focus on research on food environment in LMICs,^13^ critical research gaps exist around the foodways of indigenous communities and factors influencing them, particularly in India. These knowledge gaps limit the ability to design context-specific interventions to ensure better nutritional outcomes of vulnerable communities. Thus, obtaining knowledge on the basic elements of a community’s foodways could be crucial towards understanding their vast landscape of beliefs, customs, and traditions, necessary for ensuring food justice and better nutritional outcomes. The present study explores the foodways of Ho indigenous community of Jharkhand and describes the ways in which they maintain their traditional food and land systems, intertwined with their cultural food beliefs, customs, and traditions. Our intent was to provide a case study on one of the indigenous communities of India, who have potentially resilient traits along with rapidly transitioning food environment. Information from this study will potentially help indigenous communities globally to improve the use of their traditional food systems, and for researchers and policymakers to identify future research needs and necessary points of interventions, for better nutritional well-being of Indigenous Populations.

## Methods

### Study Design

An exploratory mixed-method study was conducted using qualitative and quantitative enquiries to gain insights into the foodways of Ho community in West Singhbhum district of Jharkhand, India. This work was part of a larger study documenting the role of IFs consumed towards ensuring food security and dietary diversity among tribal communities of Jharkhand.^14^ The data collection was conducted between December 2021 to September 2022; multiple visits were conducted to complete the qualitative and quantitative data collection.

### Study Locale and Population

The study was conducted in West Singhbhum district, Jharkhand, which has a total area of 12,805 Sq. km, spread across 18 blocks.^15^ Among these blocks, three geographically distinct blocks (namely, Sonua, Khuntpani and Chakradharpur) with high population density of Ho community, were purposively selected. Using probability proportional to size sampling, a total of ten villages were selected from Sonua, Khuntpani and Chakradharpur blocks (Supplementary Table 1).

### Study Procedures

A total of 10 focus group discussions (FGDs) (one in each village) were conducted with 5-10 adult women of mixed age groups, particularly young and old women, identified through snowball sampling. Inclusion of women facilitated rich information on food collection and preparation. Pre-tested FGD guides were utilized to capture information on the general livelihood sources, traditional farming methods, access to diverse food sources including food production and foraging, market access, social and cultural norms around foods, ways of food preservation, environmental factors, and community health. The FGDs were held in accessible areas such as Anganwadi centers (community-based centers for women and children) or in common areas in the villages like chaupal (common space in village for community discussions). Each FGD took about 45 to 60 minutes to complete.

Village transect walk interviews were conducted in all the ten villages to understand the demography (population, castes/tribes, religion), infrastructure, hygiene and sanitation conditions, livelihood sources, food access, and health and nutrition problems in the community. Key stakeholders (village leaders, and health and nutrition workers-Anganwadi workers and Accredited Social Health Activists (ASHAs)) who knew the village territory and surroundings well, participated in the transect walks. Seasonal market surveys (monsoon and winter) were also conducted in ten local markets or *Haats* (commonly accessed by participating villages) to capture the diversity of food items available at different time points in a year. Information was obtained from the food vendors on the type of food items sold (under different food groups), their varieties, and their price (in INR).

The entire data collection was conducted and supervised by the core research team which included a principal researcher and an assistant (for note taking, village mapping and recording). The FGDs and the village transect walks were primarily conducted by the core research team, with assistance from well-trained local field workers, fluent in the native Ho language. The field workers also conducted the market surveys.

### Data Analysis

The FGDs and interviews were recorded and transcribed verbatim from Ho to Hindi and then translated to English. The transcribed data were coded using Atlas.ti version 7.5.7. The data were coded using inductive approach; similar codes were identified and merged to produce relevant themes and sub-themes using thematic framework analysis. The quantitative data from market surveys were entered in MS Excel and analysed using descriptive statistics.

### Ethics Approval

The study was conducted according to guidelines laid down in Declaration of Helsinki ^16^ and the study procedures involving humans were approved by the Institutional Ethics Committee at Indian Institute of Public Health-Delhi, Public Health Foundation of India, and Department of Home Science, Lady Irwin College, University of Delhi. Administrative approvals from authorities at district level and cluster level consent from the village leaders were obtained prior to study initiation. Verbal informed consent was obtained from local market vendors. For FGDs, written informed consent was obtained from literate respondents and third-party witnessed verbal consents were sought from respondents, who could not read and write. All respondents were informed that the FGDs and interviews were being audio recorded.

## Results

**Fig. 1:**
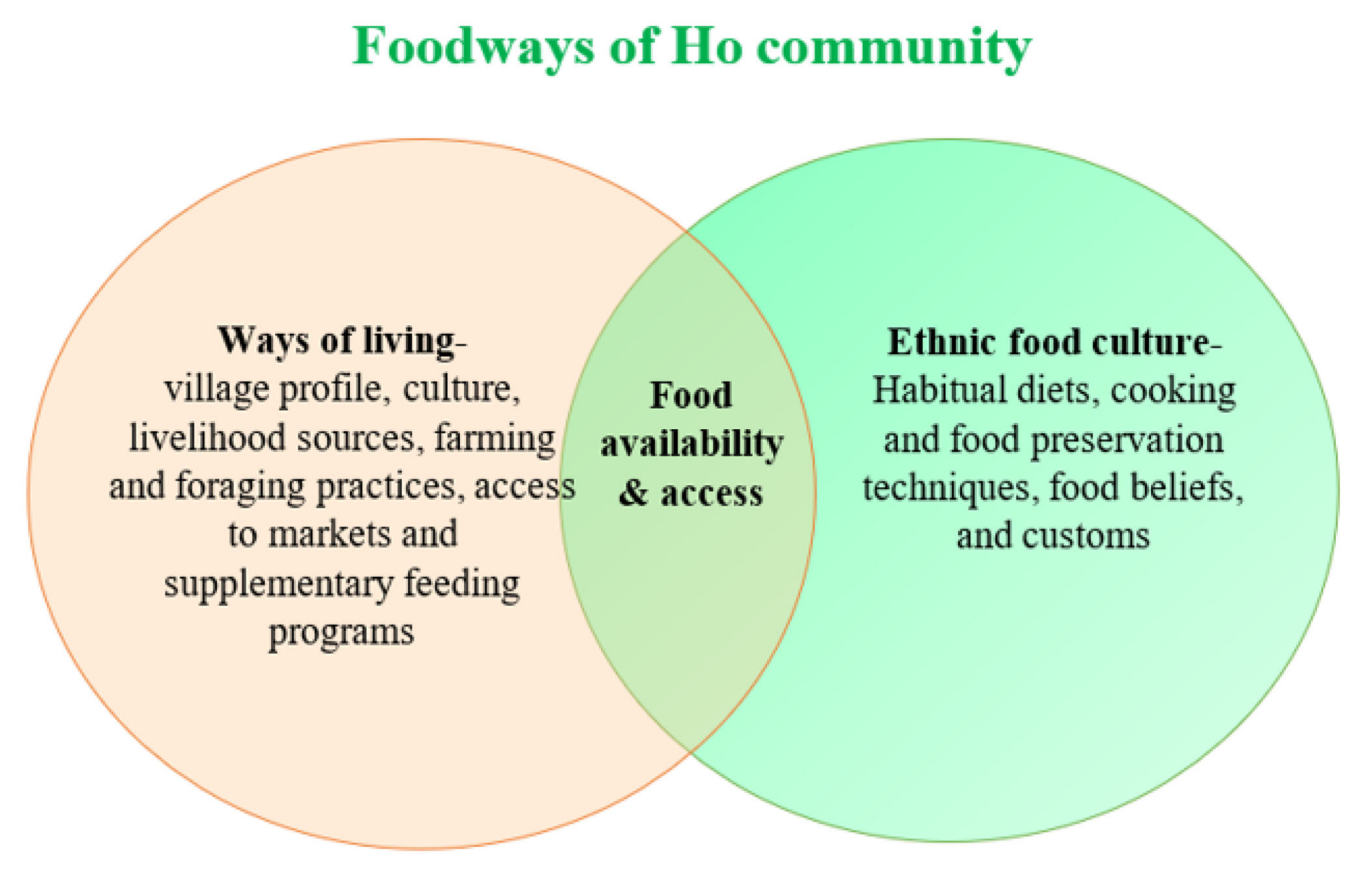
Components examined as part of foodways of Ho indigenous community, Jharkhand, India

In this section, the foodways of the Ho community are described, starting from their general ways of living, with information on their living conditions, livelihood sources, farming practices, and foraging techniques. This is followed by description of their ethnic food culture, including information on their habitual diets, food beliefs and customs (Figure 1).

### Ho Community and their Ways of Living

The Ho community are the original inhabitants of West Singhbhum district, residing in villages, situated in biodiverse terrains, surrounded by dense forests and hills. These villages comprise 150-200 households on an average, and are composed of 2 or 3 hamlets, known as ‘*Tola*’, with road connectivity to the main towns. The community usually lives in a multi-ethnic setup amidst other communities (scheduled tribes, scheduled castes, and other backward castes) and follow *Sarna* religion (i.e., worship of nature). They collect drinking water from handpump, wells and *Chuwa* (collected water source near riverside) and prefer open defecation over in-house toilet facilities. Malaria and anaemia are reportedly the common health problems. All participating villages have access to public health centres (PHCs), which provide basic facilities like immunization, maternal and child healthcare and treatment against malaria, diarrhea, fever and common cold. Some villages (*Bankitapi, Basakuti, Hatnatodong and Horlor*) have the nearest PHCs at 10-20 kilometers away, which contributes to their low utilization and the communities usually uses shared or public transport to access these facilities.

The Hos are predominantly subsistence smallholder farming communities, dependent on natural produce for sustenance and livelihood. They utilize the cultivated and wild produce for household consumption and sell the remaining produce in local markets or *Haats*. These *Haats* operate daily or on selected days of the week, situated within an average range of 1-10 km from the villages and accessed through public (bus), shared (auto) or private modes of transport (cycles, bikes).

Additionally, the community breeds livestock (such as chicken, goats, duck) as financial assets to sell them in situations of income distress. However, the income from selling of cultivated and/or wild produce in *Haats* is often not sufficient to sustain the day-to-day living expenses. Consequently, some sections of the community are transitioning towards alternate sources of livelihood like wage labouring. Migration is another key phenomenon; between the months of July to September, male members from some households finish the plantation work in the fields and temporarily migrate to different states (Maharashtra, West Bengal, Tamil Nadu, and Gujarat) in search of better work opportunities. Females also permanently migrate to Bangalore, Chennai, and Vishakhapatnam where they work in stitching centres and factories.

### Hunting and Gathering Practices

**Fig. 2:**
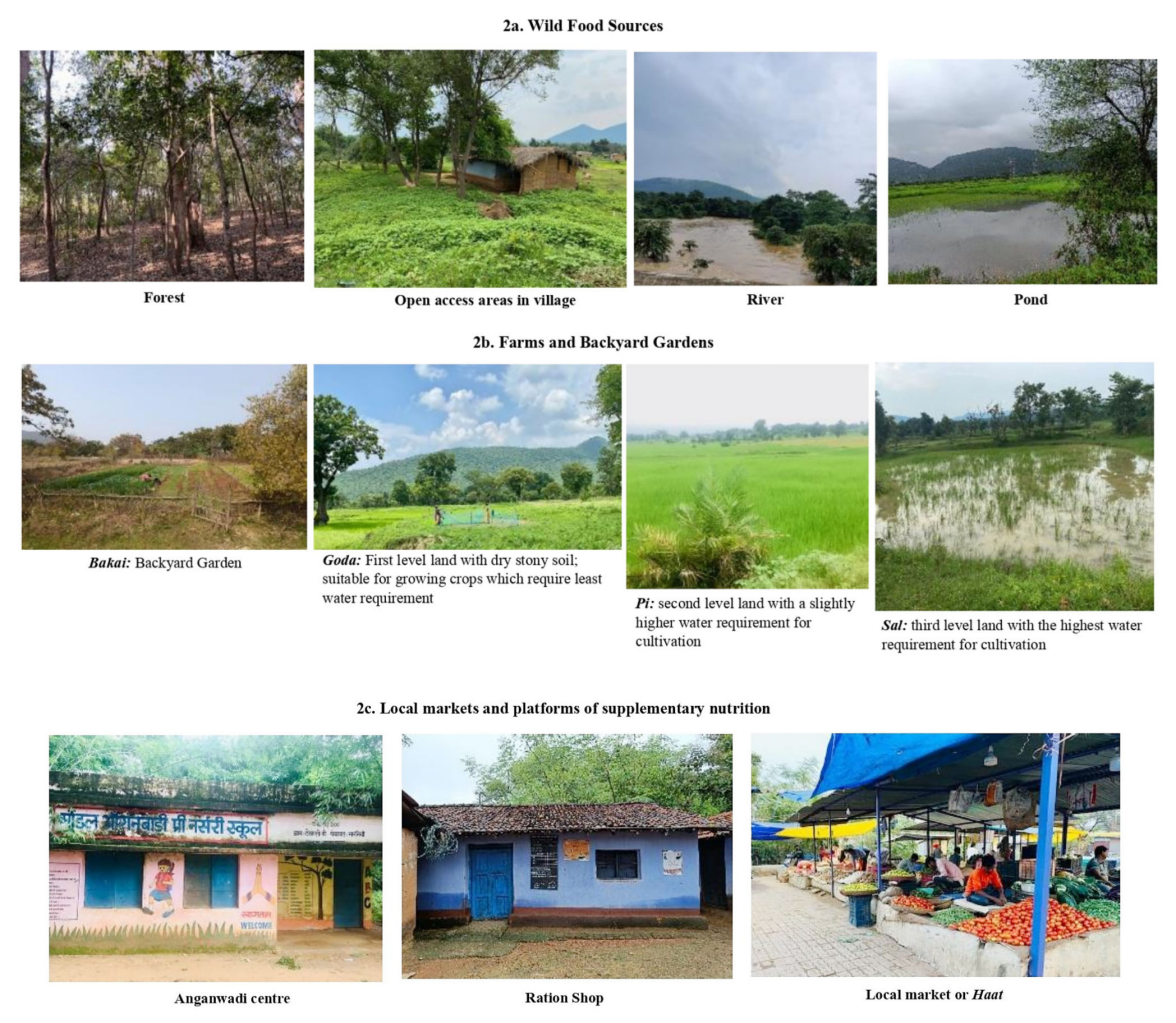
(a-c): Sources of food access in Ho indigenous community of Jharkhand

Wild habitats are a significant source of sustenance and livelihood for the Ho community. The surrounding forests, roadsides, wastelands, and water bodies (Figure 2) are accessed for collecting most of the food items including GLVs (Koinaar leaves (*Sing aa*), Katai leaves (*Sarli aa*)), mushrooms (*Pata Ud, Simdali Ud*), fruits (Tumki (*Kendu*), Burflower (*Kadam*)), tubers (*Bayang sanga, Duri sanga*), and wild game (Deer (*Silip*), Indian Myna (*Rami*)) (Table 1). Foraging is primarily done by women with frequency of collection ranging from daily during the monsoon to twice or thrice a month during winters and summers. For villages like Horlor and Keadchelam, the nearest forests are located faraway from these villages, contributing to high opportunity cost of foraging in terms of time and convenience. One woman spoke about changing priorities on food procurement: “*Yes, it’s a hassle*… *even after going there (forest) we may get it (foods) or not…sometimes need to buy it (food) from market*…”(Respondent number 1, female, study village three, Khuntpani block). The male members of the community go for hunting of wild animals in large groups, during the spring season (February-March) lasting till the early onset of summer season (April). This practise of hunting, is however, gradually declining with time, owing to the restrictions imposed by the forest department on hunting and the high opportunity cost associated with travelling, searching, and hunting the wild game.

Ho women also collect firewood from forests, for use as cooking fuel. The frequency of collection ranges from every day to 2-3 times a week across all seasons and is done discretely by the community due to the restrictions imposed by the forest department on cutting of trees. However, the lack of provision of alternative cooking fuels in most villages, renders the community helpless and solely dependent on firewood for cooking. As one community member opined “*Forest department prohibits. They don’t let us cut wood. They prohibit bringing wood for burning. We don’t get gas cylinder also so what we will do*?” (Respondent number 1, female, study village four, Chakradharpur block). Some community members also collect leaves to make disposable plates for selling in *Haats*.

### Traditional Agricultural Practices

Settled agriculture is practiced on plain farmlands, with an average farm size of 3-4 *Bigha* (traditional unit of land measurement, equivalent to 0.25 Hectares), divided into three levels: *Goda, Pi* and *Sal* (details in Figure 2). The agriculture is predominantly focused on paddy production, but the community also cultivates diverse crops including legumes, GLVs, and other vegetables on farms and backyard gardens (*Bakai*) for home consumption. These crops are cultivated in three main agricultural cycles: *Kharif* (June-November), *Rabi* (December-March) and *Garma* (summers) cycle (March-June). During the Kharif cycle, different indigenous varieties of paddy (*Koya Dhan, Bojna Dhan, Sorna Dhan, Aril Dhan* and many more) are cultivated; in the Rabi cycle, indigenous varieties of legumes (*Kansari* (khesari dal), horse gram (*Kurthi*), ricebean (*Sutri dal*)), oilseeds (flaxseeds (*Tisi*), niger seeds (*Surguja*)) and some vegetables (field beans (*Sidmi*), sponge gourd (*Nenua*), ridge gourd (*Jhinga*)) are grown. During the Garma cycle, GLVs like green amaranth leaves (*Leper aa*), sweet potato leaves (*Aadi Sanga aa*), garkha leaves (*Sirgiti aa*)), wild tubers (elephant yam (*Haathimanda*), ban-aloo (*Piske sanga*), and vegetables like ladyfinger and ivygourd (*Kundri*) are grown in Bakai or Goda lands. Table 1 lists the names of common crops cultivated in farms and Bakai.

Gender roles in farming are well-defined; men start with ploughing the fields using draft power (oxen) to prepare the land for planting of seeds, and women undertake several activities like de-weeding (i.e., removing the grass), sowing, manuring, and transplantation. The crops, once matured, are harvested by both men and women. The farming is largely rain-fed with additional dependence on ponds and rivers for irrigation and use of organic manure (cow dung). Indigenous seeds are preferred for cultivation (over hybrid ones), due to their 1) desirable organoleptic properties, 2) climate resilience, and 3) low-resource intensive nature (less requirement of farm inputs). These indigenous seeds are stored using traditional methods, as explained by one community member: “*We make “Puda*” *(small basket) out of hay. To cultivate crops next year, we tie the (indigenous) seeds in “puda” only and store it. If it is kept properly, then we can keep it well even for 5 years. During our grandfather’s time, they used to keep it even for 10 years.”* (Respondent number 1, female, study village two, Chakradharpur block). Some of the low-resource and climate-resilient indigenous crops include indigenous red gram (*Rehad*), Khesari dal, cowpea (*Barbatti*), flaxseed, ivy gourd, field beans, sponge gourd, ridge gourd, and two important indigenous varieties of paddy-*Goda Dhan* and *Dongor Dhan*. Few community members with larger landholdings, are however, shifting towards hybrid seeds for higher crop yields. They utilize the indigenous crops for home consumption and sell the hybrid farm produce in *Haats* to earn additional income.

Local organizations and initiatives by state government like JSLPS (Jharkhand State Livelihood Promotion Society), PRADAN (Professional Assistance for Development Action) and women’s self-help groups (Mahila Samuh) guide the community about farming techniques, including “Sri-vidhi”-also known as System of Rice Intensification (SRI).^1^ Although, the community has mixed reactions on this technique, as one villager commented “*Children eat 2-3 plates of that (Sri-vidhi dhaan). It has bit of vitamin. But it was not enough. The rice that we grow with “Sri-vidhi” (technique) through government, is sweet and it gets digested soon. To fill the stomach, it is eaten in more quantity (as compared to indigenous rice*).” (Respondent number 4, female, study village one, Chakradharpur block).

Climate variability in the form of erratic rainfall pattern, is being observed since the past decade, impacting the food production in different agricultural cycles. This has reportedly been leading to a shift in the sowing cycle as well as spoilage of crops, ultimately resulting in poor crop yield, particularly for paddy. For instance, one person commented, “*It is not raining timely, so farming is also not happening on time. Earlier we used to sow paddy in April, but now it reaches even June” (Respondent number 3, female, study village four, Chakradharpur block). while another person stated “We heard that earlier paddy used to be available in good quantity…heard about early times…but now in our time, paddy is not growing…well. in earlier time they used dung and compost for farming.… nowadays fertilizers are being used.… rain is not sufficient, so cultivation is not going well…*. “ (Respondent number 2, female, study village three, Khuntpani block).

### Market Access and Utilization of Supplementary Nutrition Programmes

**Fig. 3:**
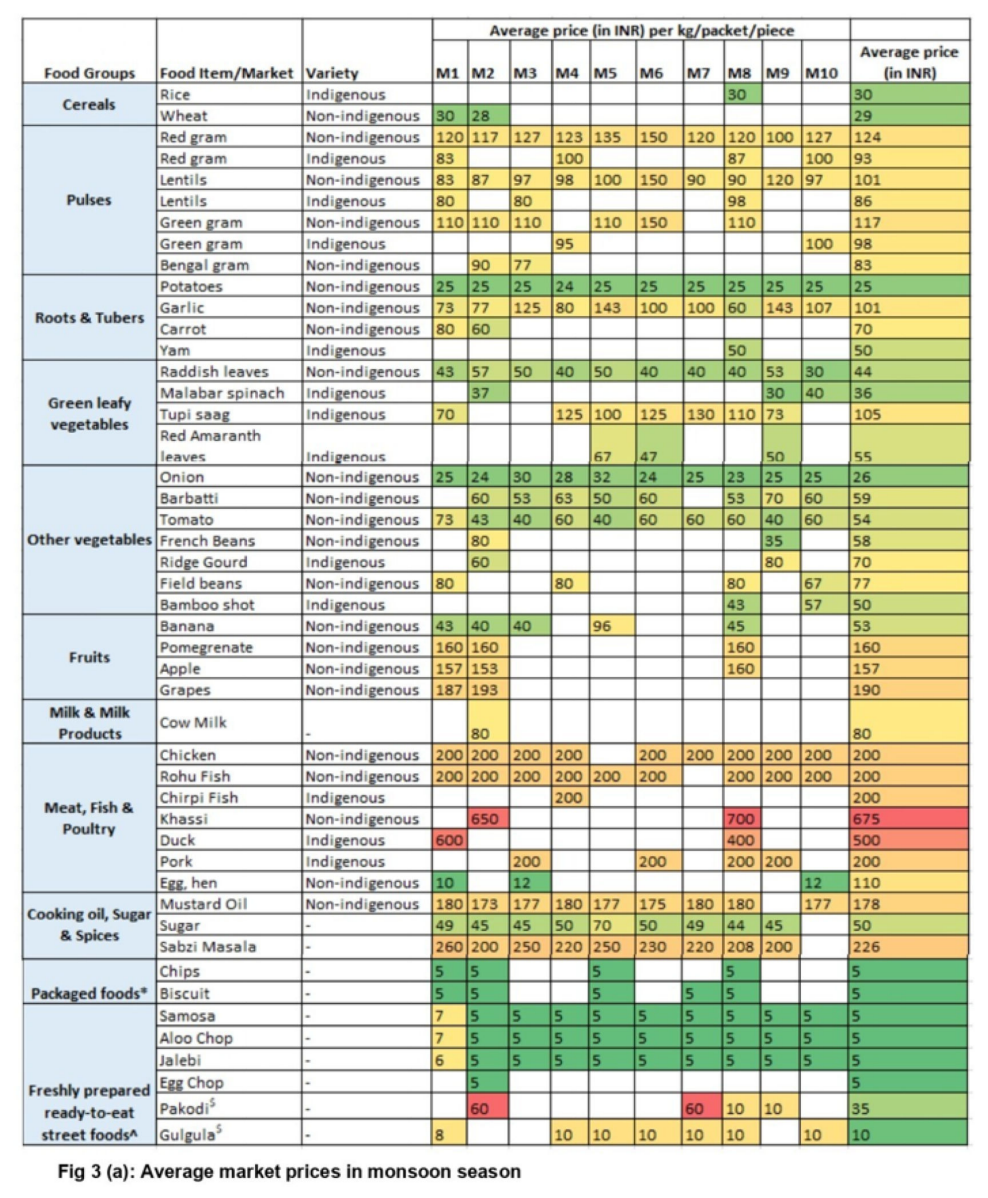

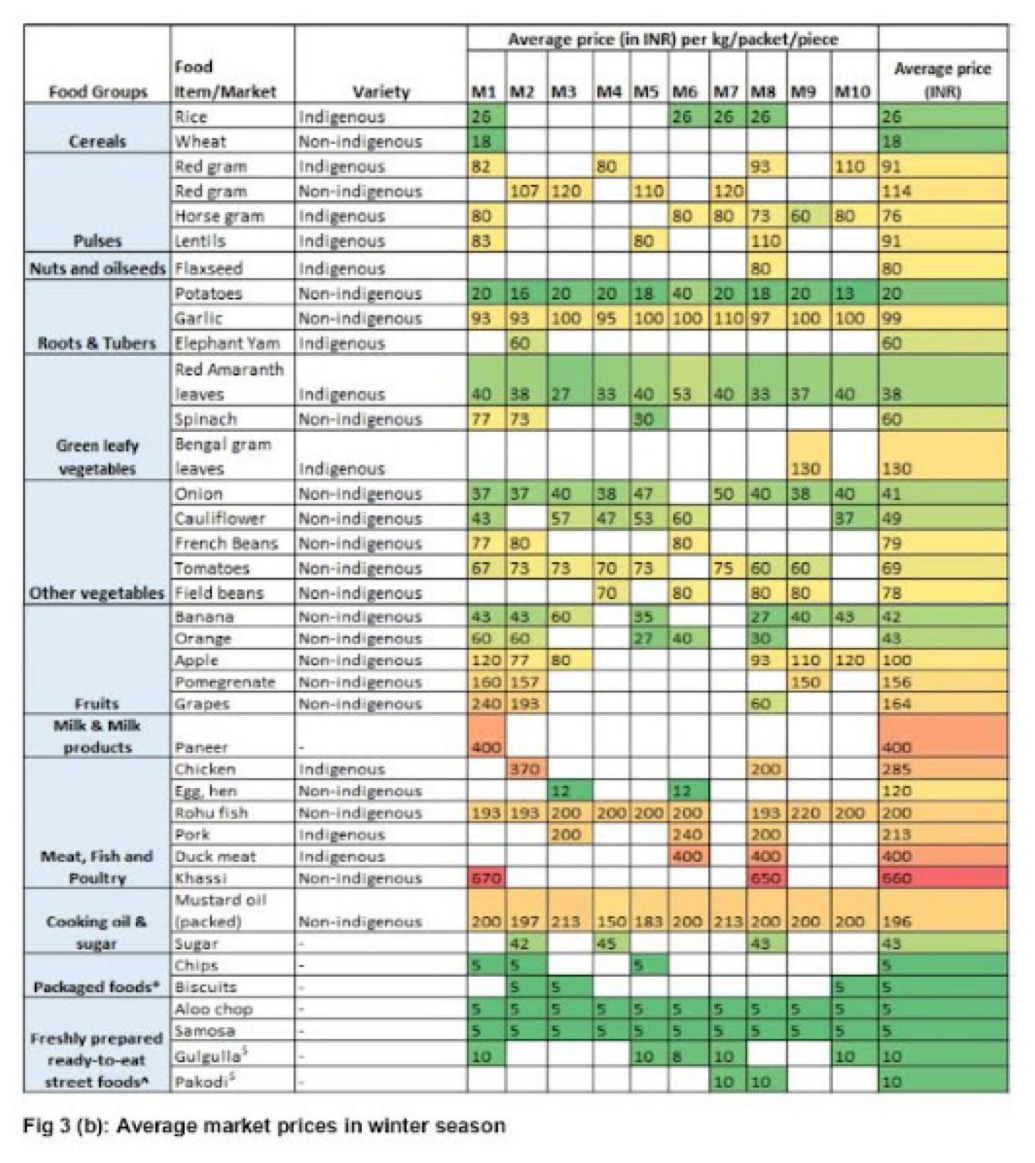
(a-b): Average prices of selected food items available in local markets accessed by Ho community *Note: M1-M10 refers to local markets 1-10; M1-Chaibasa, M2-Chakradharpur, M3-Kuida, M4-Basahatu, M5-Khuntpani, M6-Pandrashali, M7-Sharda, M8-Toklo, M9-Ulugutu, M10-Sarjomhatu The prices of cereals, pulses, green leafy vegetables, other vegetables, roots and tubers, fruits, milk products, meat, fish and poultry, cooking oil, and sugar are mentioned per kg. *The prices of packaged foods are mentioned per packet ^The prices of freshly prepared ready-to-eat street foods are mentioned per piece ($except for Pakodi and Gulgula-the prices are mentioned per plate)

A wide diversity of food items is sold in *Haats*, among which rice, potatoes, certain GLVs (red amaranth, malabar spinach, radish leaves) and vegetables (onion, cauliflower) are found to be economical (in price range of INR 20-50 per kg) (Figure 3). Pulses, flesh foods, fruits, cooking oil and spices are the costliest food items while packaged foods and freshly prepared ready-to-eat street foods are the most affordable items (prices in the range of INR 5-10 per pack/piece) across both seasons.

Government programmes like Targeted Public Distribution System (TPDS)^2^, Integrated Child Development Services (ICDS)^3^ and Mid-Day Meal Programs (MDM) (now known as PM POshan SHAkti Nirman (PM-POSHAN^4^)) are common platforms for supplementary nutrition. Through TPDS, the Ho community receives items like rice, wheat, sugar, and kerosene oil (cooking fuel) at subsidized rates and through ICDS, hot cooked meals (HCM) (for spot feeding of children at Anganwadi centres) and take-home ration (THR) are provided to pre-school children, pregnant women, and lactating mothers. Primary school going children also receive meals through MDM program.

### Ethnic Food Culture of the Ho Community Eating Habits

The food culture of Ho community depicts their closeness to nature, with IFs as an intrinsic part of their diets, representing a diversity of flavours and textures. A typical traditional Ho meal includes predominantly a carbohydrate source (*Mandi* or rice, in high amounts) eaten along with either starchy tubers or GLVs, albeit in little amounts. In breakfast, rice is replaced with Rotis (bread made of whole wheat flour obtained through TPDS) as a carbohydrate source, accompanied with only Lal Chai (black tea). The Ho food culture also includes fortnightly consumption of flesh foods, including meat of wild pig, chicken, goat and duck. Meat of animals like sheep, rabbit, deer, pigeon as well as fishes like Pool Barb, walking catfish, prawns, snails, are rarely consumed.

Despite owning cows and goats as livestock, the Ho community does not traditionally consume animal milk. Handiya or rice-beer, a traditional alcoholic beverage made from fermented rice, is consumed during ceremonies and festivals. Many households also consume it daily, usually during the morning time and some households like to consume it as part of the main meal by replacing the staple foods (rice). Preparation of Handiya is also a means of livelihood for certain households wherein the woman prepares and sells the drink in the villages or in *Haats*.

### Cooking and Preservation Techniques

The food preparation of Hos includes minimum ingredients; pulses, vegetables and meat are cooked with salt, spices (turmeric and chilly) and condiments (onion and garlic) with limited use of mustard oil. The seasonal availability of several food items encourages the community to prolong the shelf-life of these foods through common food preservation techniques like sun-drying, pickling and chutney preparations (sauces prepared from fruits/vegetables/GLVs/flesh foods, with spices and condiments). Some IFs preserved through sun-drying include 1) GLVs like katai leaves (*Sarli aa*), chimti leaves (*Mui aa*), sweet potato leaves (*Aadi Sanga aa*), drumstick leaves, colocasia leaves, pot cassia, 2) wild mushrooms (like *Pata Ud and Simdali Ud*), 3) fruits like Mahua, 4) vegetables (*Hutarba* flower, fruit peels of Ipeel/roselle leaves), and 5) small fishes (like *Chirpi*/Pothi fish). These dried food items are consumed as curries by reconstituting the dried food (in powdered form) with water; for example, *Maad Jhor*, a popular curry dish, is prepared from reconstituting dried green leafy vegetables with rice water (*Maad*) with tomato and onion as key ingredients.

Pickling of several fruits, vegetables and tubers is a common practise. Examples of pickled fruits include hog plum (*Amda*), wild yam (*Hadah*), wood apple (*Bael*), and Ipeel (fruit of Roselle leaves), GLVs like Phutkal leaves, and certain insects (like red ants). Chutneys are a common meal accompaniments among the Ho tribe. Some of the popular chutneys consumed by the Ho community include *Hauko chutney* (red ant ground using a stone with garlic, ginger and red chilli), *Ipil chutney* (fried and softened peels of the fruit of Rosella leaves, cooked with spices and made into a paste using water), *Tisi chutney* (roasted flaxseeds, grounded into powder form, mixed with rice water, red chillies, garlic and onion to form a paste) and *Amda chutney* (made of boiled Amda fruit pieces, cooked with ginger and garlic) (Figure 3). With gradual introduction of cheap ultra-processed foods and street foods in *Haats*, the community (especially the youth) is demonstrating a gradual dependence on these foods.

### Food Customs during Local Festivals

**Fig. 4:**
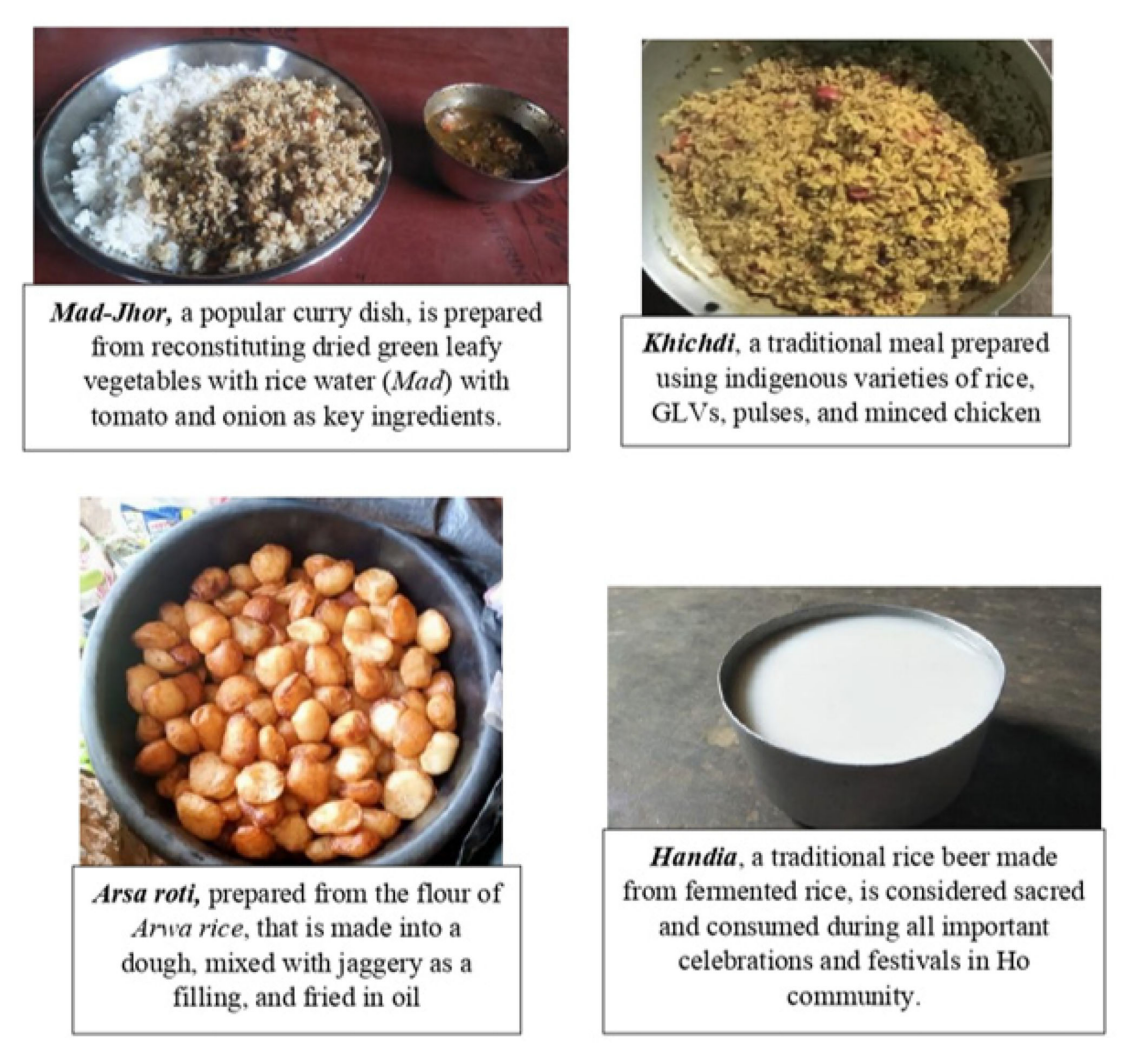
Traditional recipes of Ho indigenous community of Jharkhand

The Ho tribe celebrates four main parab or festivals throughout the year, which mark as an occasion for the community to come together and celebrate their culture and traditional cuisines (Figure 4). The most important festival, *Maagh parab*, is celebrated at the onset of spring season (March-April), which marks the completion of the agricultural cycle in the Ho community. During this festival, a traditional meal of *Khichdi* (prepared using indigenous varieties of rice, GLVs, pulses, and minced chicken), is consumed. *Baha Parab* (meaning festival of flowers) is another important festival, celebrated in mid-spring (February-March), for worshiping of new flowers of Sal and Mahua trees. The new flowers of Sal and Mahua are worshipped and consumed along with boiled fruit of Mahua. *Baha Parab* also marks the onset of mango season, which is celebrated by worshipping and consuming the first grown mango of the season. Lentils (*Masoor Dal*) is a popular dish in this festival, with fresh or dried indigenous fishes as key ingredients. Hairo Parab is celebrated in August, marking the end of the sowing cycle of the paddy crop. This festival is celebrated by worshipping the goat, which is then sacrificed and consumed, along with *Arwa Roti* (chapatis made from flour of *Arwa rice*, a special indigenous rice variety) and curry made using Amaranth leaves (Leper aa). *Jomnama Parab* is the last festival of the year, celebrated in October, to worship the newly harvested paddy crop. In this festival, a famous rice-based dish known as ‘*Arsa roti*’ is worshipped by all family members and consumed along with an indigenous fish or meat of chicken/goat. Additionally, the Ho community also prepares rice flakes from the newly harvested rice, and worship it with Soso leaves (leaves of wood apple plant).

### Local Food Beliefs and Taboos

Like other communities, the Ho community also believes in certain food related restrictions and taboos (Table 2). The pregnant and lactating women are prohibited from consuming a diet rich in variety of foods like fresh fruits, vegetables, GLVs and flesh foods, under a common perception that consumption of these foods is bad for new-born child’s health. Due to these restrictions, the new mothers consume a diet comprising only plain boiled rice and pulses, devoid of oil, which grossly comprises their nutritional quality and calorie density throughout the day.

## Discussion

The Ho community of Jharkhand resides amidst a biodiverse environment, yet have suboptimal living conditions owing to poor water, sanitation, and hygiene (WASH) practices and low access to healthcare services. Although majority of the community is engaged in smallholder farming, a section of the community is now transitioning to other forms of livelihood. The Ho traditional foodways are based on foraging, hunting, and crop cultivation, and make good use of the available ecosystem resources through use of indigenous foods, seeds, and organic manure. They have unique food traditions and cultures, yet their meals lack variety in terms of the foods consumed.

Although Hos cultivate varieties of vegetables and GLVs in their backyard gardens, their agriculture is predominantly based on paddy production, utilized mainly for home consumption. As reported in qualitative enquiries, the community is not able to earn desired profits through their smallholder farming systems, exacerbated by ongoing impacts of climate variability on the cropping cycle and yields. This is leading to rural-to-urban migration. Studies have highlighted the multiplier effect of the job-driven out migration of the rural workforce, linked with globalization of their food choices, on the overall integrity of traditional food systems of indigenous communities.^20^ According to a geospatial evaluation of various datasets in Jharkhand, about 73% of the land in West Singhbhum district has high potential for agroforestry, which may perhaps boost the local economy, promote ecological sustainability and improve income and livelihood in the Ho community.^21^ This potential is however underexplored and perhaps the role of MGNREGA could be crucial for promoting livelihood security in such regions through creation of jobs and assets for improved water security, soil conservation and higher land productivity.^21^

Despite Ho community’s access to a wide variety of IFs, the high opportunity cost of accessing these foods from wild habitats may have future implications on their consumption. This has also been reported in other indigenous communities of Jharkhand.^6,7^ A high dependence on market-procured food items was observed, with a particular preference towards cheap ultra-processed foods, and other freshly prepared and processed street foods. This dietary shift could be attributed to the easy availability and affordability of ultra-processed foods in *Haats*, as compared to the nutrient-dense food items, as highlighted in our study. This is a typical feature of nutrition transition, where traditional foods are increasingly replaced with convenience foods (rich in sugar, salt and fats) that are easily accessible through globalization of markets.^22^ Similar trends of nutrition transition are being observed in indigenous communities residing in India and other parts of the world, thus contributing to a triple burden of malnutrition.^23,24^ The rural indigenous communities, in particular, perceive the market foods as ‘aspirational’, and traditional foods as ‘poor man’s food’; this trend may significantly violate the food sovereignty of Indigenous Peoples, leading to a loss of self-determination over their traditional food systems.^25^

The study found that Ho community is surrounded by diverse food sources, yet their meals are predominantly rice-based, with little quantities of pulses or vegetables. Several potentially micronutrient rich foods ^26^ are accessed and/or grown by the households, yet their consumption is not frequent, which may be attributed to factors rooted in their ways of living. A similar nutrition paradox has been reported in other indigenous communities of Jharkhand, highlighting the poor utilization of traditional foods in these communities.^6,7^ Energy poverty (in terms of the use of unclean cooking fuel) could be one of the factors, that adds to the women’s time and burden of fetching firewood from the forests, who are already burdened with multiple household responsibilities like foraging of wild produce, contribution in agricultural activities, and food processing and preparation activities. Scientific evidence reveals that the several responsibilities on rural women may not only add to their stress and work burden but could also have implications on their dietary intake, due to time trade-offs associated with household food preparation, food access and production activities.^27^ Further, the existence of various food taboos, especially for vulnerable groups (women, children, diseased individuals) is perhaps leading to poor consumption of nutritious foods at critical phases of life, with potential implications on dietary intake and nutritional status.

The study also highlighted other indirect factors that may compromise the health and nutritional status of Ho community. The Ho tribe was found to have poor WASH practices in terms of unsafe drinking water and preference towards open defecation (despite having toilets constructed within the house). While detailed reasons were not explored in this study, the poor WASH practices in communities residing in low-resource settings may be attributed towards their lack of awareness regarding the health hazards associated with open defecation, as well as the absence of environmental, institutional, and social support for switching towards desirable practices of using toilets.^28^ Additionally, poor access to the primary health care owing to their distant location is leading to sub-optimal utilization of government health services within the community. Similar issues have emerged in other studies on indigenous communities of India,^29,30^ thus highlighting the poor uptake of government health services by the indigenous populations. Further, the routine consumption of traditional alcoholic beverages (like Handiya) may not only lead to potential health risks but may also be detrimental towards ways of life and social architecture of the community. It is thus vital to align the long held cultural food taboos and traditions, while scientifically validating the benefits of traditional foodways to create a balanced approach towards strengthening of traditional food systems of Ho community.

## Conclusion

The Ho community of Jharkhand is intricately connected to its ethnic food culture and traditions and reside in villages nestled among dense forests and foothills. Despite living in harmony with nature, their living conditions are poor, and their meals are not diverse. Thus, the traditional foodways of Hos are potentially under threat due to gradual livelihood and nutrition transitions. It would thus be crucial to skilfully engage the community in programs and interventions, wherein their local traditional ecological knowledge could be effectively tapped for ensuring better food production and utilization practices. Future research directions should be focused on an in-depth understanding of the nutritional value of local flora and fauna accessed by this community and their contribution to food security and dietary diversity. Efforts are desirable to support the vulnerable indigenous smallholder farmers through access to relevant information and technology, enhanced value chain development for local plant food resources, and integration of farm produce with supplementary food programs for demand creation and monetary benefits. Alongside these approaches, behaviour change communication strategies should highlight the nutritional importance of traditional foods. This is critical in checking the growing dependence on energy-dense nutrient-poor diets. The preservation of Ho community’s sociocultural values is vital for promotion of their traditional foodways which can support bio-cultural knowledge, food justice and improved nutrition.

## Supplementary Material

Supplementary Table

## Figures and Tables

**Table 1:** Types of foods accessed from wild and cultivated food sources by the Ho community, Jharkhand, India

Food sources	Types of indigenous foods accessed
**Wild habitats** Forests	
	**Green leafy vegetables:** Koinaar leaves (*Sing aa*), Katai leaves (*Sarli aa*), Dheniani leaves (*Rimil Tundu), Bilay Moo aa, Oae manda saag* **Other vegetables:** Spine gourd (*Kakrol*), Cassia Indigo flowers (*Hutarba*), Kutma (*Anjed Jo*) **Mushrooms:** *Potkeh/Rugda, Pata Ud, Simdali Ud, Pual Chattu/Busu Ud, Gitil Ud, Sosoye* Ud, Hende Ud **Roots and Tubers:** *Bayang sanga, Duri sanga* **Fruits:** Hog Plum (*Amda*), Tumki (*Kendu*), Burflower (*Kadam*), Indian Plum (*Mirle*) **Wild game:** Deer (*Silip*), Wild pig (*Birsikri*), Rabbit (*Kulhe*), Pigeon (*Dudulum*), Indian Myna (*Rami*), House sparrow (*Dedem*), Red ant (*Kurkur/Hauko*), Hornet and Wasp (*Tumbli*)
Open spaces (roadsides, wastelands)	**Green leafy vegetables:** Pot Cassia (*Kayur aa*), Amaranth leaves, green (*Leper aa*), Amaranth leaves, red (*Lal Bhaji*), Ponnagini (*Garundi aa*), Punarnava leaves (*Khapra saag),* Tupi aa, Dah Janum aa **Mushrooms:** *Tumbe Ud, Kadhaye Ud, Bunum Ud* **Fruits:** *Bael,* Gular (*Aain Jo*), Kusum (*Baru*), Barhar (*Dahu*) **Wild game:** Spotted Dove (*Putem*), Quail (*Bater*), Honeybee larvae (*Maddhumakkhi*)
Ponds/lakes /rivers	Pool Barb (*Chirpi Hayi*), Spotted snakehead (*Chodha Hayi*), Freshwater eel (*Dondo Machli*), Freshwater Mud eel (*Koocha Hayi*), Snail (*Genda*), Bayara Machli, Walking catfish (*Magrayi machli*)
**Farmlands** *Goda*	
	**Cereals:** *Goda Dhan* (indigenous paddy variety), Sorghum (*Chauli Gangai*) **Pulses:** Horse gram (*Kudhti*), Black gram (*Biri Dal*) **Green leafy vegetables:** Garkha leaves (*Sirgiti aa*), Colocasia leaves (*Kacchu/Saru aa*), Chota Tupi aa, Sweet potato leaves (*Aadi sanga aa*) **Other vegetables:** Field beans (*Sidmi*), Sponge gourd (*Nenua/Pullu*), Sonpu flower (*Sutri ba*) **Roots and Tubers:** Desi Oal (*Hadah*),
Pi	**Cereals:** *Lal Dhan, Koya Dhan, Aril Dhan, Jarli Dhan, Wheat (Gehun*) **Green leafy vegetables:** Water spinach (*Kalmi saag*), Sunsuni leaves (*Chatom aa*)
Sal	**Cereals:** *Hende Dhan, Dansar Dhan, Dudukulum Dhan, Kausum Dhan, Dongor Baba*
Bakai (Backyard	**Pulses:** Rice bean (*Sutri Ramba*), Cowpea (*Ghangra*) **Green leafy vegetables:** Drumstick leaves (*Mulge aa*), Ashgourd leaves (),
Garden)	Malabar Spinach (*Pui saag*), Roselle leaveas (*Ipeel aa*) **Other vegetables:** Ivy gourd (*Kundri*), Ridgegourd (*Junni*), Gamarsimbi (*Malhan/Chayipi*) **Roots & Tubers:** Elephant yam (*Hathi Manda*)
**Livestock**	Goat (*Bakri*), Sheep (*Bhaid*), Wild Hen (*Jangli Murgi*)

**Table 2:** Food taboos in the Ho community, Jharkhand, India

Prohibited foods	Who avoids the foods?	Beliefs
Snail (*Genda*), wild mushrooms (particularly *Pual Chattu*) and Bengal gram (*Chana Dal*)	Pregnant women	Consumption of these foods leads to brain development disorders in new-born babies
Papaya	Pregnant women	Consumption of papaya leads to abortion of the baby
GLVs	Lactating women	Consumption of GLVs by a lactating woman transfers worms from the mother’s milk to the child’s stomach, leading to diarrhoea.
Mango, Tamarind and Hog Plum (*Amda*)	Lactating women	Consumption of tangy fruits like Mango, Tamarind and Amda after the delivery, reduces the effect of medicines prescribed to a new mother
Spicy curries (including vegetables and flesh foods)	Lactating women	Lactating mother should consume a plain diet, devoid of spices
Pumpkin (*Kakru*), Bottlegourd (*Lauki*), Ivygourd (*Kundri*), Ridgegourd (*Jhinga*)	Sick people	Consumption of vegetables that grow as creepers, makes the illness spread in the body in a similar manner to how a creeper vegetable grows and spreads on the ground.

## Data Availability

Not applicable

## Footnotes

1 A technique in which two plants are situated at 20-50 cm, requiring less irrigation water, and producing higher yield per unit field

2 Targeted Public Distribution System (TPDS) is a national food security scheme in India, under which, major food commodities like rice, wheat, sugar, salt, and/or kerosene oil are distributed to poor households at subsidized rates^17^

3 Integrated Child Development Services (ICDS) is a government program which provides nutritional meals, preschool education, primary healthcare, immunization, health check-up and referral services to children under 6 years of age and their mothers ^18^

4 PM POSHAN scheme, previously known as Mid-Day Meal program, is a centrally sponsored scheme that provides free mid-day meal on working days for school children studying in primary and upper primary classes in government schools ^19^
