# Supplementary material for: Exploring the traditional foodways for nutritional well-being amongst vulnerable communities: Insights from Ho indigenous community of Jharkhand, India
Source: Curr Res Nutr Food Sci. Author manuscript; Available in PMC 2025 Mar 13. (PMC7617473; doi:10.12944/CRNFSJ.12.2.14)
Supplement: Supplementary Table [file EMS203057-supplement-Supplementary_Table.pdf]

**Supplementary Table 1. List of selected study villages in Sonua, Khuntpani and Chakradharpur blocks, West Singhbhum, Jharkhand, India.**

| S.No. | Block 1: Sonua | Block 2: Khuntpani | Block 3: Chakradharpur |
|---|---|---|---|
| 1 | Punipada | Loharda | Komae |
| 2 | | Basakuti | Toklo |
| 3 | | Keadchalam | Hatnatodong |
| 4 | | Baripi | Bankitapi |
| 5 | | Horlor | |
